# Impact of Rare Earth Elements on CaCO_3_ Crystallization: Insights into Kinetics, Mechanisms, and Crystal Morphology

**DOI:** 10.1021/acs.cgd.3c00858

**Published:** 2023-12-30

**Authors:** Luca Terribili, Remi Rateau, Adrienn M. Szucs, Melanie Maddin, Juan Diego Rodriguez-Blanco

**Affiliations:** Department of Geology. School of Natural Sciences, Trinity College Dublin, Dublin D02PN40, Ireland

## Abstract

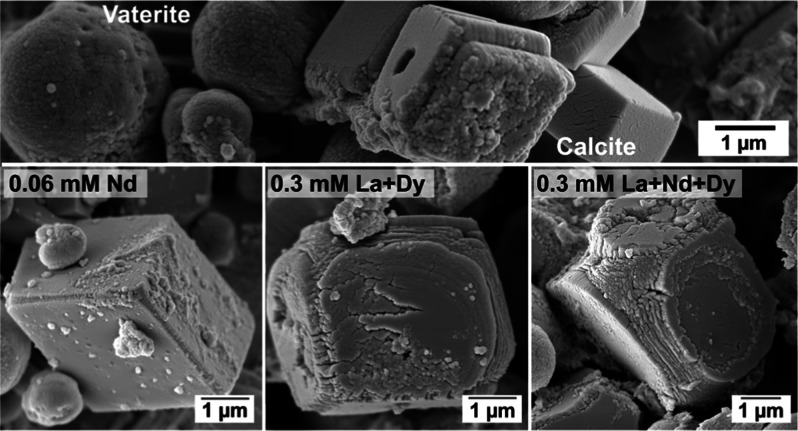

This study investigated the crystallization kinetics and mechanisms of calcium carbonate (CaCO_3_) in the presence of rare earth elements (REEs) including lanthanum (La), neodymium (Nd), and dysprosium (Dy). Through a comprehensive approach utilizing UV–vis spectrophotometry, powder X-ray diffraction, and high-resolution electron microscopy, we examined the effects of REEs on CaCO_3_ growth from solution at varying concentrations and combinations of REEs. Our findings highlight that even trace amounts of REEs significantly decelerate the rate of CaCO_3_ crystallization, also leading to alterations in crystal morphology and mechanisms of growth. The impact of REEs becomes more pronounced at higher concentrations and atomic mass, although the potential formation of poorly ordered REEs carbonate precursor phases can result in a decrease in the REE^3+^/Ca^2+^ ratio, influencing the crystallization rate of CaCO_3_. Vaterite and calcite were identified as the main crystallized polymorphs, with vaterite exhibiting distinct growth defects and calcite developing complex morphologies at higher REEs concentrations and an internal architecture suggesting a nonclassical growth route. We propose that REEs ions selectively adsorb onto different calcite surfaces, impeding growth on specific sites and resulting in intricate morphologies.

## Abstract 2

The effects of REEs (La, Nd, and Dy) during the crystallization of calcium carbonate (CaCO_3_) were examined using a combination of UV–vis spectrophotometry, powder X-ray diffraction, and scanning electron microscopy. Results reveal that trace amounts of REEs decelerate the rate of CaCO_3_ crystallization, leading to alterations in crystal morphology and mechanisms of growth. Vaterite and calcite exhibit respectively growth defects and complex morphologies suggesting a nonclassical growth route. All these effects become more pronounced at higher concentrations and atomic mass of REEs.

## Introduction

Rare earth elements (REEs) are a group of 15 metallic elements on the periodic table, called also lanthanides, at which are sometimes added scandium (Sc), and yttrium (Y)^1^ due to their similar properties and the fact that they occur in the same ores.^2^ REEs are generally classified into two subgroups: light rare earth elements (LREEs), including La to Eu (atomic numbers 57–63), and heavy rare earth elements (HREEs), including Gd to Lu (atomic numbers 64–71) and Y due to its similar chemical properties.^3,4^

REEs have exceptional electromagnetic properties, making them critical to high-tech industries and clean energy applications^2,5−8^ (i.e., wind turbines and rechargeable electric car batteries^9,10^) and for this reason, their demand is expected to continuously increase in the near future.^9^

Despite their importance, REEs are also considered critical resources due to the risk of supply disruption and potential shortages in the near future^11^ and both the European Commission^12^ and the US Department of Energy^13^ consider them to be critical or near-critical in terms of supply risk.^14,15^ Specifically, Dy, Tb, Eu, Nd, and Y are at high risk of supply disruption in the medium to long term.^13,15,16^

The world’s REEs reserves are located in a few principal countries: China, Brazil, Vietnam, Russia, and India.^17^ China currently has 42% of global REEs reserves and a significant percentage of the world’s HREEs reserves^7^ while Brazil and Vietnam follow with approximately 16.9 and 16.7% of total reserves, respectively.^2,7^

REEs sources are bastnäsite [(REE)CO_3_(OH)], monazite [(REE)PO_4_], and xenotime [(Y,REE)PO_4_]. The first two are enriched in LREEs while xenotime is the main source of HREEs and Y.^2,18−20^ Processing these minerals is very complex, requiring specific processing methods^20,21^ which are environmentally harmful and often inefficient.^21−23^

Therefore, it is imperative to find more sustainable ways of obtaining REEs in the future and to better understand the genesis of their ore deposits, including REEs-rich fluids and mineral interaction processes.

Calcium carbonate (CaCO_3_) minerals are common in nature and play an important role in biomineralization^24^ but can be also found in many different hydrothermal environments (i.e., white smoker chimneys, hydrothermal veins and carbonatite deposits^25−27^). They have important applications both for large-scale technological challenges (e.g., carbon capture and storage, nuclear waste disposal)^24^ and everyday applications.^28^ CaCO_3_ can form five polymorphs, three anhydrous (calcite, vaterite, and aragonite) and two hydrated (monohydrocalcite, CaCO_3_·H_2_O, and ikaite, CaCO_3_·6H_2_O). Furthermore, it can also form a hydrated and poorly ordered amorphous solid phase, amorphous calcium carbonate (ACC) (CaCO_3_·*n*H_2_O, *n* < 1.5).^29−31^

Previous experimental studies have shown that REEs are strongly adsorbed on the surface of Ca–Mg–Sr carbonates and often substitute for Ca^2+^ ions within these minerals.^32−37^ Incorporation of REEs into calcite at ambient temperatures is important as they can be used as geochemical tracers in terrestrial and marine waters.^37,38^ Moreover, the study of REEs incorporation into minerals is essential because REEs exhibit similar crystallochemical properties to actinides in the oxidation state of +3.^37,39−42^ Poorly ordered precursor ACC and metastable phases such as ikaite, monohydrocalcite, and vaterite are known to uptake foreign ions from solution,^30,43−45^ but their ability to uptake REEs has not been studied accurately. Despite their significance, current knowledge regarding the uptake of REEs during mineral formation and recrystallization processes, such as aragonite–calcite, vaterite–calcite, and monohydrocalcite–aragonite transformations, is limited.

This study aims to shed light on the impact of REEs on the kinetics and mechanisms of CaCO_3_ crystallization at the micro- and nanoscale. The existing literature on this topic is very limited, with most studies focusing primarily on the effect of La^3+^ ions during the crystallization of calcite.^46−48^ Further research on the interaction between REEs and carbonates during their crystallization is crucial for gaining a better understanding of the impact of REEs on the early stages of mineral formation.^49^ REEs primary deposits are carbonatites, igneous rocks containing more than 50% carbonate^50,51^ of which some aspects of the origin remain uncertain.^51,52^ These deposits are complex systems ruled by many physicochemical parameters (e.g., temperature, pressure, chemistry of solids and fluids) (e.g.,^51,52^) and one of the major problems in the exploration and exploitation of REEs contained within them is the lack of understanding of the crystallization mechanisms and pathways of REEs carbonates.^49^ Therefore, this study will contribute significantly to advancing our knowledge of this broad field.

The effects of individual and combined REEs in the kinetics and crystallization mechanisms of CaCO_3_ polymorphs from solution at ambient temperature were investigated through a set of solution experiments at starting low supersaturation conditions (below ACC solubility). The crystallization kinetics of CaCO_3_ were followed in situ and real-time, and the solids were characterized to determine how single and multiple REEs affect their polymorph selection and crystal morphology.

## Material and Methods

Crystallization experiments were carried out by mixing at a ratio of 1:1 4 mM Na_2_CO_3_ solution with 4 mM CaCl_2_ solution (pure or doped with different concentrations of REEs) at ambient temperature and under constant gently stirring conditions. For that purpose, CaCl_2_ was doped with 0.06, 0.1, 0.15, 0.2, 0.25, and 0.3 mM of different REEs alone or in combination (La, Nd, Dy, La + Nd, La + Dy, Nd + Dy, La + Nd + Dy). All solutions containing multiple REEs were prepared with equimolar solutions, and the concentration indicated refers to the total combined concentration of REEs. All solutions were prepared with reagent grade chemicals and pure deionized water.

Crystallization reactions were followed in situ and real-time with time-resolved UV–vis spectrophotometry, examining the change in solution absorbance (turbidity), after the methods of Tobler et al. (2014, 2015, 2016)^45,53,54^ and Rodriguez-Blanco et al. (2014).^55^ For this purpose, equal volumes (1 mL) of doped CaCl_2_ and Na_2_CO_3_ solutions were mixed in a 5 mL cuvette and continuously stirred. The UV–vis spectrophotometer (Ocean Optics) was set to measure the absorbance at 450 nm wavelength at a time interval of 1 s. Experiments were repeated from 3 to 5 times to ensure good reproducibility.

In all our real-time UV–vis experiments, we are going to assume that CaCO_3_ nucleation begins simultaneously with the absorbance process, though there is a possibility that a very small fraction of the particles may crystallize just before the initial rise in absorbance.

Any error resulting from this assumption is expected to be proportionally consistent across all experiments regardless of the aqueous chemistry. Similarly, the point where the absorbance reaches its maximum is going to be considered to correspond to the moment when equilibrium is attained concerning the primary crystallized CaCO_3_ polymorph(s).^45,53,54^

Replica experiments were repeated in larger reactors in order to obtain a larger quantity of solid samples for characterization with powder X-ray diffraction (XRD) and scanning electron microscopy with energy dispersive spectroscopy (SEM–EDS). In this case, the experiments were carried out by mixing 100 mL solution of CaCl_2_ doped with REEs and 100 mL Na_2_CO_3_ solutions, gently stirring, and collecting solid samples after 20 min. The resulting solutions were immediately filtered using a vacuum filtration system with 0.2 μm polycarbonate filters and rinsed with isopropanol to prevent potential recrystallization or formation of other solids from any residual interstitial water and then quickly dried in air.^53,56^

The crystalline nature of the solid samples was determined by powder XRD. The samples were analyzed with a Bruker D5000 powder X-ray diffractometer (Cu Kα radiation, 0.02 step^–1^ from 5 to 70° in 2θ at 5 min^–1^) located at Trinity Technology and Enterprise Centre (Dublin). Identification of crystalline phases present in the samples obtained was carried out with the Bruker DIFFRAC.EVA software in combination with the ICDD Powder Data File (PDF-4, The International Centre for Diffraction Data). Pattern-matching refinement, quantification of crystalline phases, and analysis to determine the Bragg peaks broadness (full-width half-maximum, fwhm calculation) were carried out with the Rietveld refinement software TOPAS.^57^

Images of the precipitates were obtained with SEM–EDS to study potential changes in the morphology and size of the crystalline phases. In order to prepare the samples for SEM characterization, these were placed on mounts and coated with carbon, using a Cressington 208 carbon High Vacuum Carbon Coater. SEM–EDS analyses were conducted using a TIGER S8000 FEG-SEM operating under high vacuum conditions and equipped with two Oxford X-Max 170 mm^2^ EDS detectors running the Oxford AZtec analysis software. The analyses were carried out using a beam current of 300 pA and an accelerating voltage of either 5 kV, for detailed imaging, or 20 kV, for EDS analysis at the iCRAG Lab at Trinity College Dublin. Multiple Point EDS analyses and compositional maps were carried out on the samples to determine the atomic % of La, Nd, Dy, Ca, and O present in the crystalline phases. The standard used for the SEM–EDS analysis was a cobalt wire with a purity of 99.995%. The particle size distribution in the samples was measured with ImageJ Software.^58−60^

Quantification of the reaction extents was used to provide information on the rate of crystallization using the empirical Avrami equation^61^
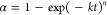
1where *k* is a rate constant, t is time, α is the fraction crystallized, and *n* is a constant that depends on the transition mechanism. Rewriting the Avrami equation gives
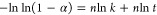
2

The reaction with kinetics that conform to this equation give a straight line when –ln ln(1 – *y*) is plotted against ln t.^62−64^ The empirical parameter *n* value is given by the value of the slope, which can be used to compare the reaction mechanism. Parallel lines indicate a constant value of *n*, suggesting that the reaction mechanism is the same. The intercept on the *y*-axis gives the value of *n* ln *k*, by which the *k* value can be determined.^62^

## Results

The combination of UV–vis spectrophotometry, powder XRD, and SEM–EDS allowed the understanding of the kinetics and mechanisms of CaCO_3_ crystallization in the presence of the REEs and revealed that the REEs affected the crystallization kinetics and morphology of the precipitated CaCO_3_.

**Figure 1:**
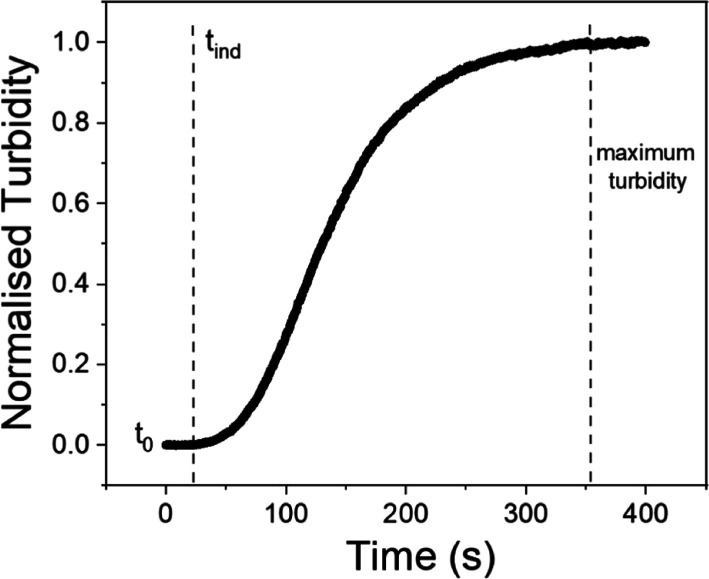
Normalized turbidity profile for the crystallization reaction of CaCO_3_ in the pure system pure system. The mixed solutions are 4 mM Na_2_CO_3_ and 4 mM CaCl_2_ without any dopant. The *t*_0_ and *t*_ind_ indicate, respectively, the beginning of mixing of the solutions and the end of the induction time. In the curve it is possible to appreciate the evolution of the crystallization rate *k*: initially it increases rapidly, decreasing afterward until reaching of a maximum of turbidity.

In all experiments, the reaction exhibited a consistent general pattern characterized by an initial sluggish stage, followed by an acceleration, and ultimately reaching a maximum absorbance (Figure 1).

**Figure 2:**
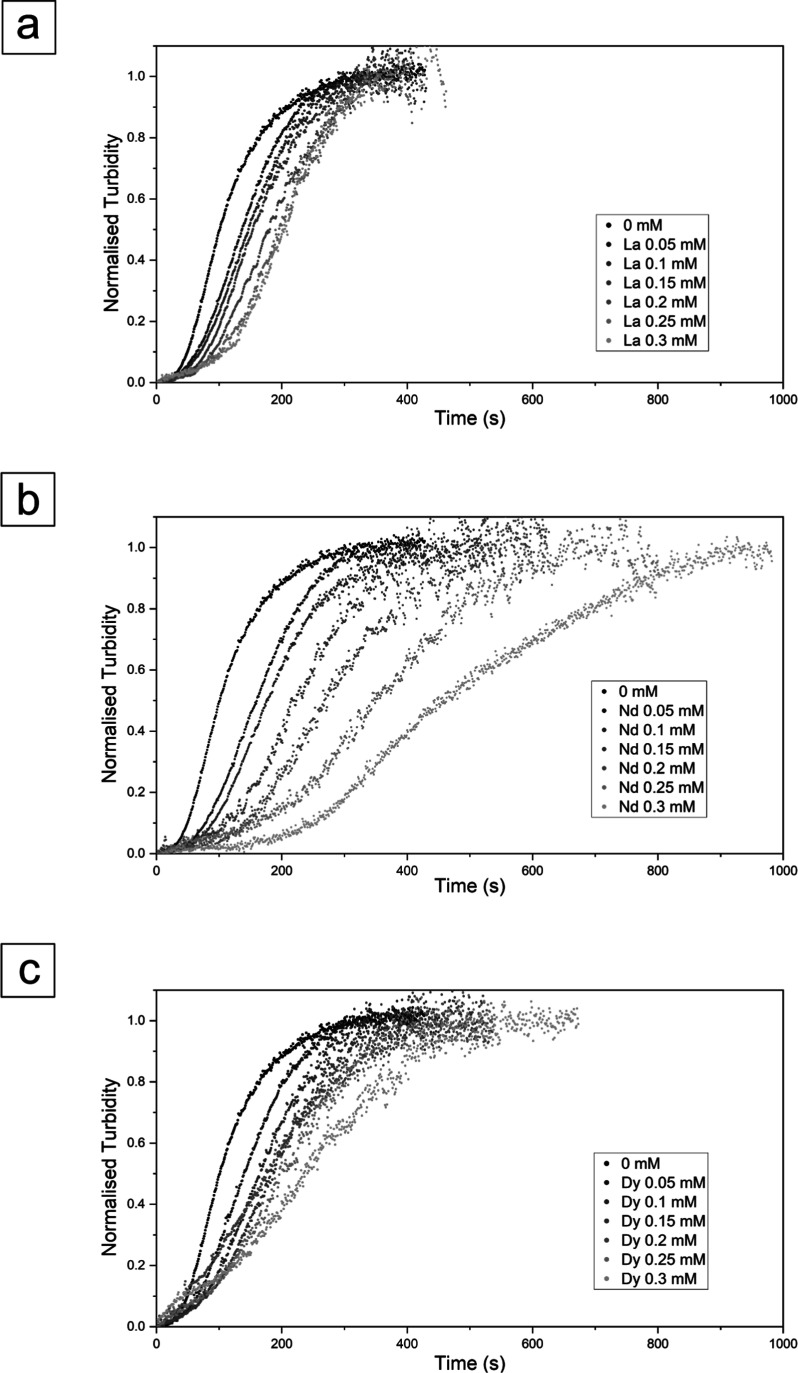
Normalized turbidity graphs showing the effect of single REEs during the crystallization reaction of CaCO_3_. The graphs refer to (a) the CaCO_3_ system with La, (b) Nd, and (c) Dy with different concentrations from 0 to 0.3 mM.

The crystallization rate of CaCO_3_ was the fastest in the absence of REEs (0 mM; pure system; Figure 1; Figure 2). Under these conditions, the reaction was initiated after a brief induction period of approximately 20 s, and the absorbance reached its maximum value after 340 s. The addition of REEs to the experiments led to a decrease of the kinetics of CaCO_3_ crystallization (Figure 2). The effect was proportional to the concentrations and atomic numbers of the REE(s) used.

In experiments where CaCO_3_ crystallized in the presence of single REEs (La, Nd, Dy), the induction times and crystallization rates were slower, leading to longer times to reach maximum absorbance and complete crystallization compared to the pure system. With regard to these experiments, La had the shortest induction time, ranging from ∼30 to 65 s, Nd had the longest, ranging from ∼40 to 150 s, while Dy showed an induction time comprised from ∼40 to 80 s (Table 1). The end of the crystallization reaction occurred approximately between 365 and 380 s for La, 385 and 930 s for Nd, and 380 and 615 s for Dy, respectively, for experiments carried out at 0.06 and 0.3 mM. These results suggest that La has the least effect on calcium carbonate crystallization, followed by Dy and Nd, with the strongest effect (Figure 2).

**Table 1:** Values of the Induction Times Reported for the CaCO_3_ Crystallization Experiments Carried out in the Presence of Different Concentrations of REEs

	induction time (s)						
REEs	0 mM	0.06 mM	0.1 mM	0.15 mM	0.2 mM	0.25 mM	0.3 mM
La	23	29	34	41	52	58	66
Nd	23	38	45	56	94	125	146
Dy	23	41	44	48	51	55	78
LaNd	23	31	33	35	37	38	41
LaDy	23	36	38	40	42	45	48
NdDy	23	38	43	47	52	64	73
LaNdDy	23	38	45	46	55	63	75

**Figure 3:**
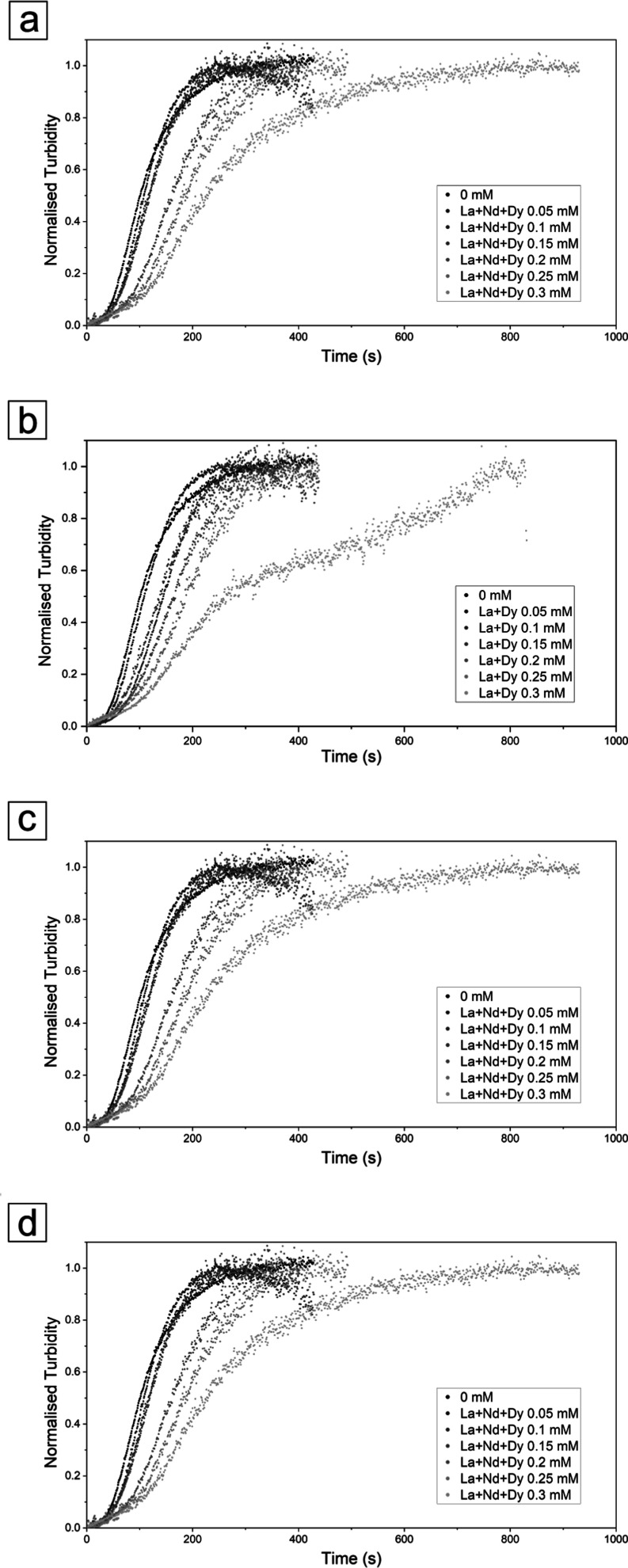
Normalized turbidity graphs showing the effect of multiple REEs during the crystallization reaction of CaCO_3_. The graphs refer to (a) the CaCO_3_ system with La + Nd, (b) La + Dy, (c) Nd + Dy, and (d) La + Nd + Dy. All multiple REEs solutions have equimolar concentrations, with total combined concentrations of REEs ranging from 0 to 0.3 mM.

The multi-REEs crystallization experiments, which involved different combinations of two or all three REEs, exhibited a similar trend to the single-REEs reactions, displaying a slowdown in the crystallization kinetics compared to the pure system. This trend was seen to increase with increasing atomic mass and concentration of the REEs in the starting solution. Induction times were longer than in the pure system, ranging from ∼30–40 s in La + Nd and ∼40–70 s in Nd + Dy (at 0.06 and 0.3 mM concentrations). The La + Dy experiment had an induction time of ∼40 s at both concentrations (Table 1). The La + Nd experiment reached its maximum crystallization at ∼290 s (0.06 mM) and ∼450 s (0.3 mM), the La + Dy at ∼250 s (0.06 mM) and ∼790 s (0.3 mM), the Nd + Dy at ∼280 s (0.06 mM) and ∼850 s (0.3 mM), and the La + Nd + Dy had induction times ranging from ∼40–70 s and reached its maximum crystallization at ∼250 s (0.06 mM) and ∼810 s (0.3 mM). Overall, the crystallization of CaCO_3_ in the presence of different combinations of REEs, La + Nd had the least impact on the process, followed by La + Dy, with Nd + Dy producing the greatest effect (Figure 3).

A minor variation within this general pattern was solely observed in experiments with the highest REEs concentrations (e.g., 0.25 and 0.3 mM Nd, Dy, or Nd + Dy) (Figure 2a,b; Figure 3c) and consisted of a slight increase in turbidity occurring a few seconds after mixing of the aqueous solutions. This increase, ranging between α values of approximately 0–0.015, lasted for less than 100 s prior to the main increase in absorbance.

**Figure 4:**
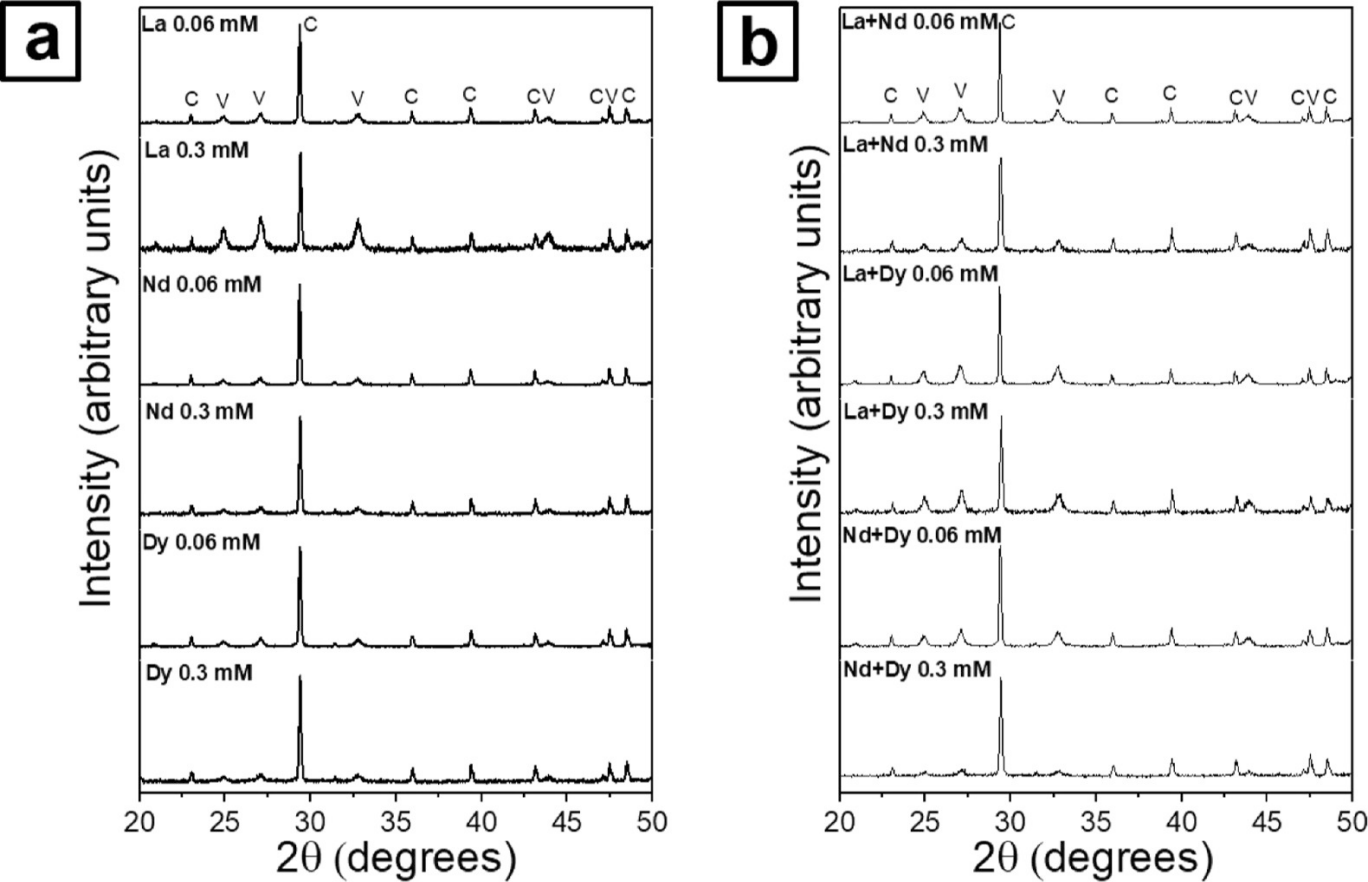
Powder XRD patterns of the experiments in the presence of (a) single-REEs and (b) multi-REEs at both low (0.06 mM) and high (0.3 mM) concentrations.

All experiments were translated to the formation of CaCO_3_. In the pure system, the crystallized CaCO_3_ polymorphs were calcite (95%) and vaterite (5%). The addition of REEs, either alone or in combination, did not affect polymorph selection but favored the crystallization of vaterite over calcite, with some differences in weight % trends (Table SI-1 and Figure SI-6). No other crystallization phases were observed (Figure 4).

In single-REEs experiments, calcite wt % was generally higher at lower REEs concentrations (0.06 mM) than at higher concentrations (0.3 mM), with the highest wt % reported in the presence of Nd and the lowest in the presence of La. In the multi-REEs experiments, the opposite trend was observed: at lower REEs concentrations (0.06 mM) calcite wt % was mainly lower than at higher concentrations (0.3 mM). In this case, the highest wt % was seen in the presence of Nd + Dy while the lowest was seen in the presence of La + Nd + Dy. Additionally, an increasing trend in the wt % of vaterite was observed, which was proportional to the atomic mass and number of REEs present in the solution. In the La + Nd + Dy combination experiments (Figure SI-8), vaterite was the most abundant phase with a wt % of around 70%. In all experiments, the Rietveld refinement did not show any changes in the unit cell parameters of the vaterite and calcite polymorphs. However, it showed that the Bragg peak broadness (i.e., fwhm) was wider for vaterite than for the calcite, thus indicating a smaller crystallite size in the vaterite compared to the calcite.

**Figure 5:**
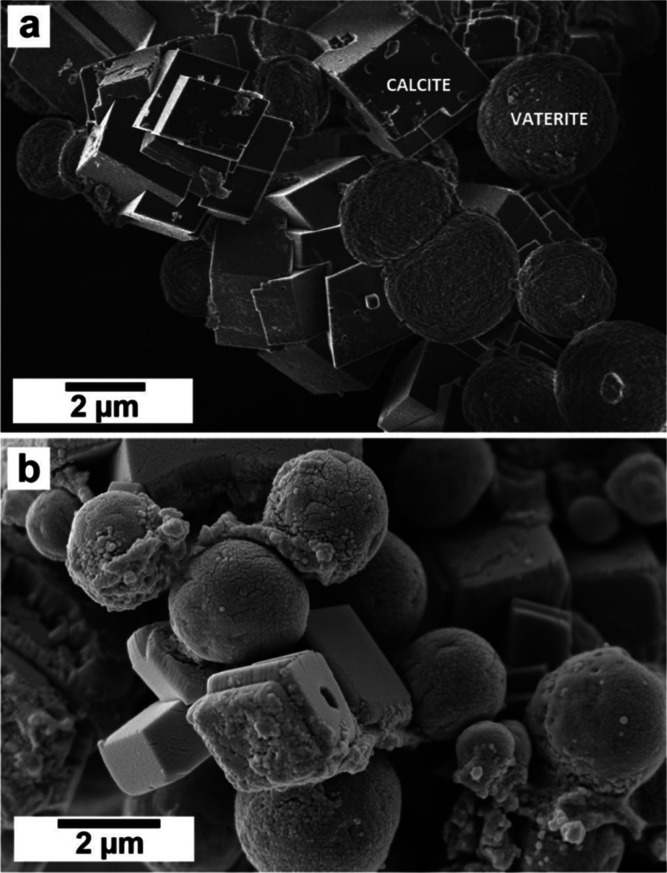
(a) SEM image showing vaterite and calcite crystallized in absence of REEs ions (pure system) with their usual morphologies, respectively spherical and rhombohedral. (b) SEM image showing vaterite and calcite crystallized in the presence of low concentration of REEs (0.06 mM La) showing spherical and rhombohedral morphologies with small defects and imperfections.

SEM imaging revealed that REEs significantly affected the morphology of calcite and vaterite (Figure 5). In the pure system and at small concentrations of dopants, calcite crystallized as rhombohedral prisms, and vaterite showed its common spherulitic morphology, consisting of spherical aggregates made of tiny nanocrystals (Figure 5a).

**Figure 6:**
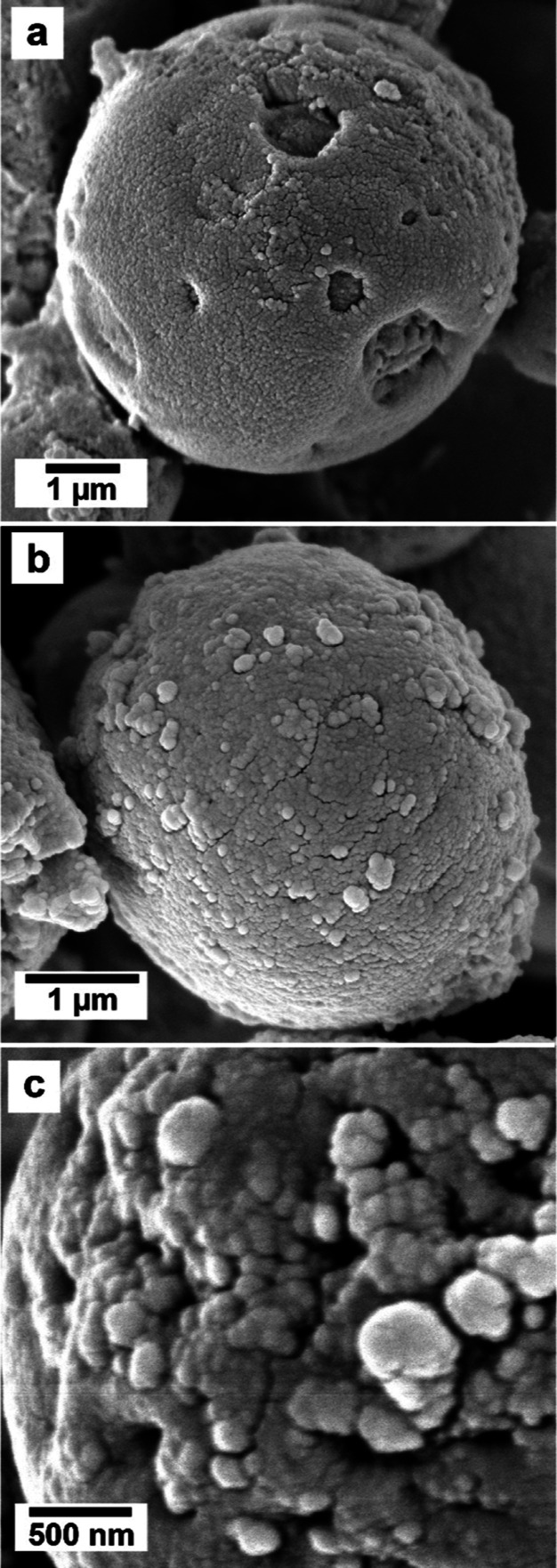
SEM images of vaterite crystallized in the presence of REEs showing an increase in the roughness of the surface and the presence of holes. (a) Holes on a spherical vaterite (Nd 0.06 mM), (b) rough surface (La + Nd + Dy 0.06 mM), and (c) detail of the surface of vaterite showing nm-sized crystals and multiple growth defects (La + Nd + Dy 0.06 mM).

However, vaterite formed under the influence of REEs developed minor defects and imperfections (rough surfaces and small holes on the surface) that increased proportionally to the concentration and the atomic mass of the specific REE/s in solution (Figures 5b and 6).

**Figure 7:**
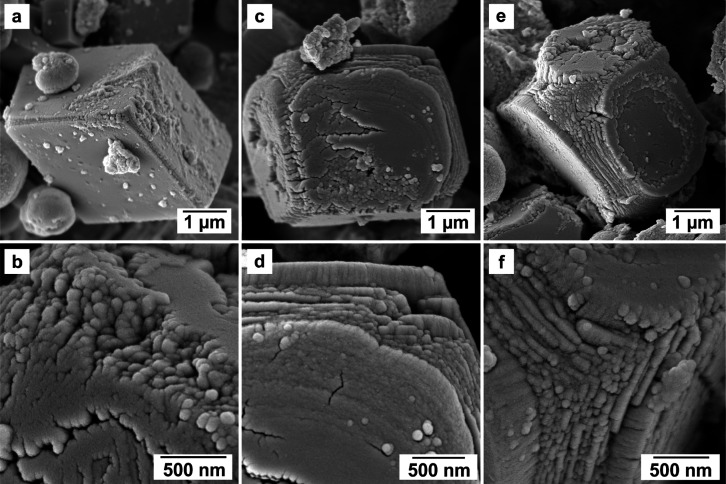
SEM images showing the increasing effects of REEs ions in solution on calcite crystals. Above: (a,b) light effects (Nd 0.06 mM), (c,d) medium effects (La + Dy 0.3 mM), and (e,f) strong effects (La + Nd + Dy 0.3 mM). Images (b–f) show details at the nanoscale.

**Figure 8:**
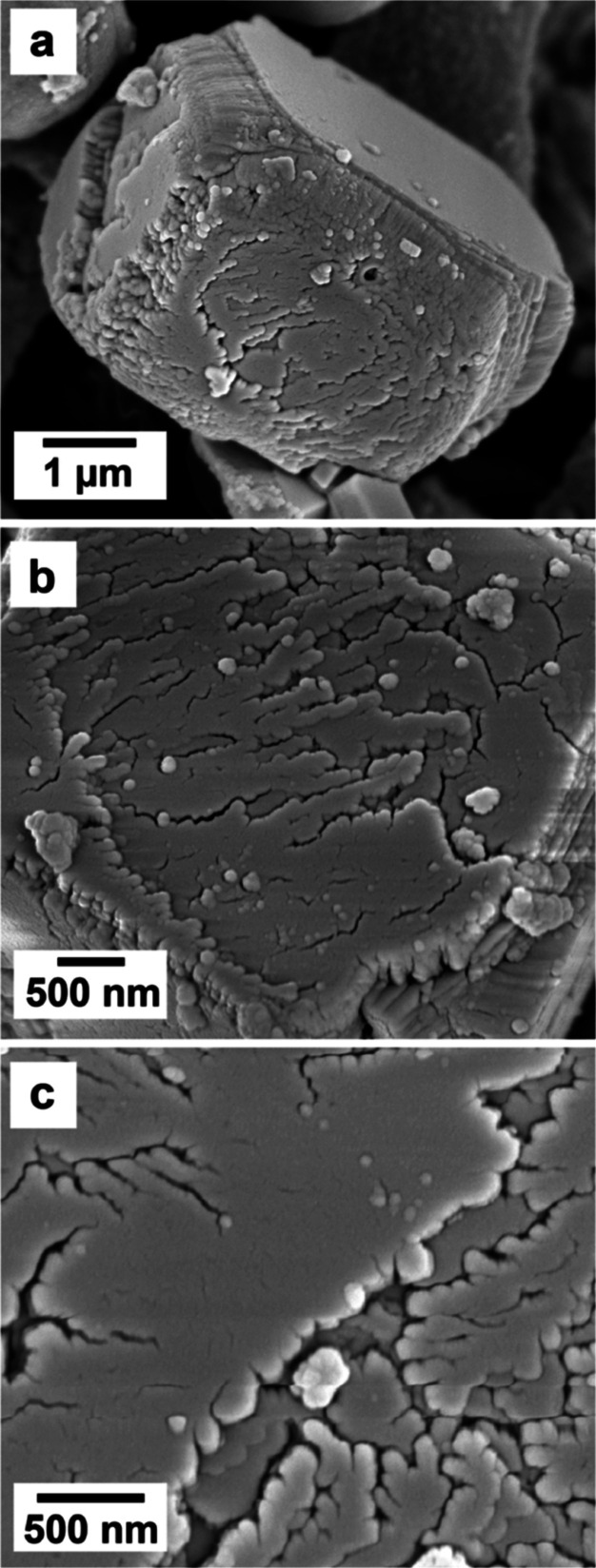
SEM images showing details of the calcite (104) face obtained in the La + Dy 0.3 mM experiment (a), which consist of nanodomains that grow following the [010] direction (b), resulting in a nonuniform surface architecture with numerous growth defects at the nanoscale (c).

While vaterite showed its usual spherulitic morphology in all of the experiments with some variations, calcite morphologies turned out to be highly dependent on the concentration and the atomic mass of the specific REEs in solution. In the pure system, calcite showed a classical rhombohedral morphology (Figure 5a). At low concentrations and low REEs atomic masses, calcite consisted of rhombohedral single crystals with growth imperfections in the edges and corners as well as local defects like small fractures on the surfaces (Figures 5b and 7a,b). At higher REEs concentrations and REEs masses, calcite did not always grow as single crystals but developed rough surfaces with numerous defects and imperfections (Figure 7). In many cases, the surface architecture and/or internal part of the calcite crystals consisted of nanodomains ∼ 100 nm in size, stacked on top of each other, which grew following the [010] direction on the (104) face (Figure 8). In some cases, this mineral seemed to consist of a thin single crystal core encased in a polycrystalline shell (Figure 7e,f; Figure 8).

The average sizes of calcite and vaterite remained consistent across all concentrations in the experiments. The differences between the average sizes in the single and multi-REEs experiments are very small, and thus, it was not possible to see any trend related to differences in REEs concentration or atomic mass. The average sizes of the vaterite spherical nanoaggregates were 2–3 μm, and calcite was overall slightly larger, with sizes between 3 and 4 μm.

**Figure 9:**
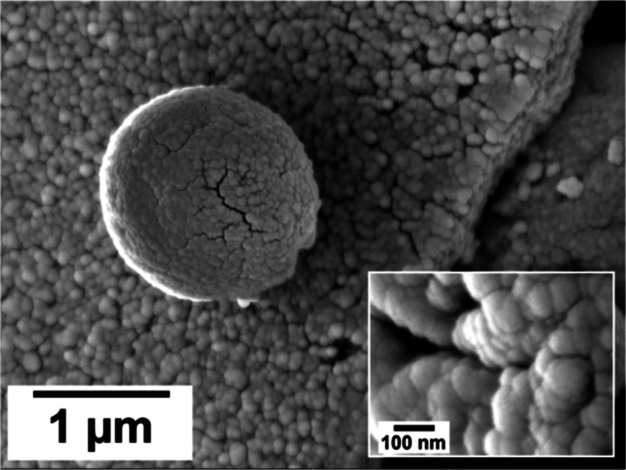
High resolution SEM image showing the amorphous poorly ordered precursor phase obtained in the experiments using the heaviest REEs (Nd and Dy) at the highest concentration (0.25 and 0.3 mM Nd, Dy, or Nd + Dy). This phase consisted of tiny spheres of few nanometres in size. The large sphere in the centre corresponds to a vaterite crystal.

In addition to calcite and vaterite, the samples obtained from the heaviest REEs (Nd and Dy) in the highest concentration experiments (0.25 and 0.3 mM Nd, Dy, or Nd + Dy) revealed the presence of an additional phase along with calcite and vaterite in the SEM images. This phase looked homogeneous in low resolution images (Figure 9), but the higher resolution images showed that it consisted of tiny spheres of few nanometers in size, located between the crystals and, sometimes, partially covering them (Figure 9).

**Figure 10:**
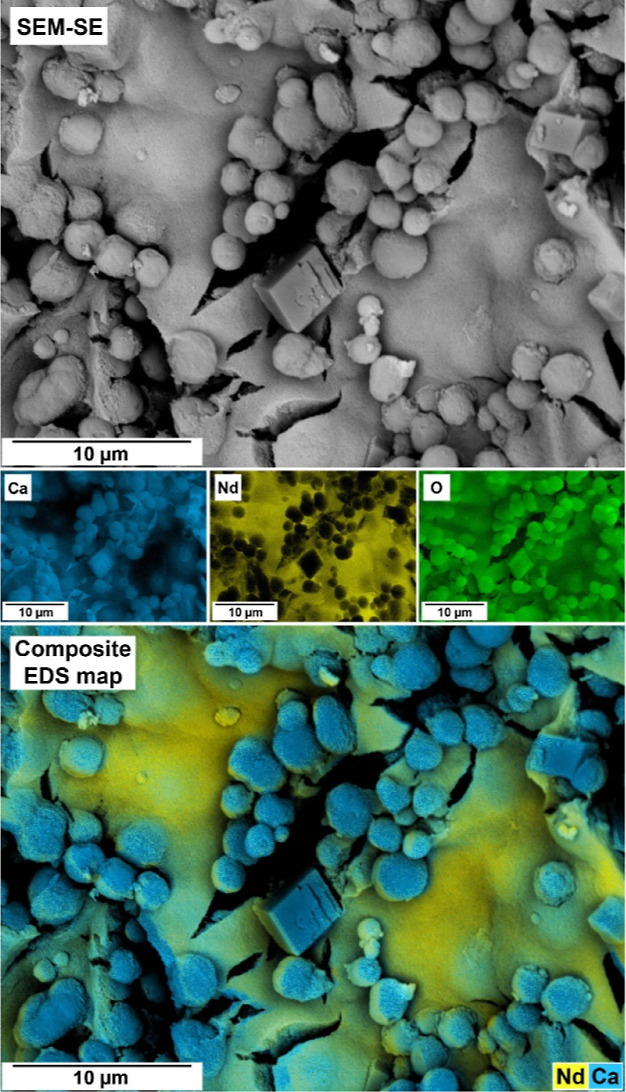
SEM image, EDS maps (Ca, Nd, and O), and EDS composite map showing a sample obtained from an experiment with 0.3 mM of Nd. Vaterite and calcite are visible in the image along with an amorphous Nd- and Ca-bearing phase.

The EDS analysis carried out on this material showed high REEs/Ca ratios compared with the CaCO_3_ polymorphs (Table SI-2). The absence of Bragg peaks indicating any additional crystalline phases coexisting with vaterite and calcite and the composition of this phase derived by EDS make it consistent with a poorly ordered precursor phase similar to the amorphous precursor REEs carbonate phases observed by Rodriguez-Blanco et al. (2014)^55^ and Vallina et al. (2013, 2015)^65,66^ in crystallization from solution experiments (Figure 10).

Despite the percentage of the amorphous phase being small, it was still possible to detect it using the XRD (Figure SI-7).

## Discussion

Our results demonstrate that REEs ions in solution, alone or in combination, can influence the mechanisms and kinetics of CaCO_3_ crystallization, as well as the resultant morphologies of the CaCO_3_ polymorphs. These effects are mainly dependent on the number of REEs, their concentration, and their atomic mass.

The pattern of the turbidity profiles obtained by using UV–vis spectrophotometry was generally sigmoidal in all of our experiments. After an induction time, the turbidity gradually increased until reaching a maximum, which indicated the end of the primary crystallization reaction. This turbidity profile is similar to other profiles produced during the crystallization of calcite in the absence of an ACC precursor.^45,67^ To get reaction rates (*k*) the patterns of turbidity were normalized through the maximum turbidity value observed for each experiment and then fitted to a JMAK (Johnson–Mehl–Avrami–Kolmogorov) particle nucleation model, which is based on the Avrami equation.^67,68^ Although this model was originally applied to solid-state phase transformations, it has also been used to fit other nucleation and growth processes exhibiting similar sigmoidal profile curves, such as crystal formation from solution and melts (e.g., refs (68)–^69^^70^). Each turbidity profile obtained in this study did not properly fit to the JMAK model using one single *n* and *k* value, giving as a result low *R*^2^ (<70%). However, it was possible to fit all turbidity profiles with two *n* and *k* values obtaining good *R*^2^ (>98%; see Table 2).

**Table 2:** Values of Crystallization Tates *k*_1_ and *k*_2_ Obtained by Fitting the Turbidity Profiles to a JMAK Model^a^

		concentration (mM)						
	*k* (×10^–3^ s^–1^)	0	0.06	0.1	0.15	0.2	0.25	0.3
La	*k*_1_	9.006	5.251	4.268	5.481	5.223	1.784	1.737
	*k*_2_	8.091	6.208	5.816	5.481	4.790	4.423	4.404
Nd	*k*_1_	9.006	5.382	4.121	1.913	2.308	0.485	2.088
	*k*_2_	8.091	5.365	4.858	3.889	3.224	2.407	1.815
Dy	*k*_1_	9.006	5.459	1.838	2.194	1.982	1.396	0.661
	*k*_2_	8.091	6.011	5.167	4.651	4.632	4.152	3.494
LaNd	*k*_1_	9.006	6.044	8.478	5.572	5.733	3.958	4.391
	*k*_2_	8.091	7.158	8.240	6.827	6.533	6.055	5.426
LaDy	*k*_1_	9.006	8.665	5.090	0.566	1.808	1.107	0.343
	*k*_2_	8.091	7.839	6.026	6.252	5.270	4.582	3.217
NdDy	*k*_1_	9.006	7.799	6.792	3.105	4.160	1.576	0.170
	*k*_2_	8.091	7.667	6.629	5.033	4.907	3.411	2.623
LaNdDy	*k*_1_	9.006	8.189	7.381	6.819	3.673	3.508	2.418
	*k*_2_	8.091	8.110	7.384	7.265	5.425	4.621	3.870

a Each turbidity profile fits to this model using two *n* and *k* values. The first *n* and *k* values correspond to the initial part of the turbidity profiles (α ≃ 0–0.07) while the second correspond to the main part of the turbidity profile up to the end of the primary crystallization reaction (α ≃ 0.07–1). The indicated concentration in solutions containing multiple REEs refers to the total combined concentration of REEs.

The first *n* and *k* values correspond to the initial part of the turbidity profiles comprised between α ≃ 0–0.07, which corresponds to early stages of the crystallization reaction. The second *n* and *k* values correspond to α ≃ 0.07 to the end of the primary crystallization reaction. These two different growth rates indicate that the crystallization of CaCO_3_ in the presence of REEs follows a multistage pathway with at least two different mechanisms of crystallization. The first part of the reaction tends to be slower compared to the second one in the experiments with single REEs and in those with combined REE at high (>15 mM) concentrations and it likely consists of the classical formation of crystalline nuclei that grow very little as a consequence of the REEs inhibiting crystal growth. In comparison, CaCO_3_ crystals in the absence of REEs grew at a faster rate from the very beginning of the crystallization reaction. In all cases, when the −ln ln(1 – *y*) values for all reactions are plotted against ln t, we observe parallel lines for the *k*_2_ value in all experiments and also for the *k*_1_ values, with the exception of the ones performed at the highest concentrations (Figures SI-2–SI-5). This indicates that the main crystallization mechanism remains the same regardless of the concentration and type of REEs used in the experiments. We consider that the slight drift in the higher concentration experiments during the earliest stages of crystallization (Figure SI-2 and SI-4) could be a consequence of the precipitation of a poorly ordered precursor shortly after mixing the aqueous solution.

**Figure 11:**
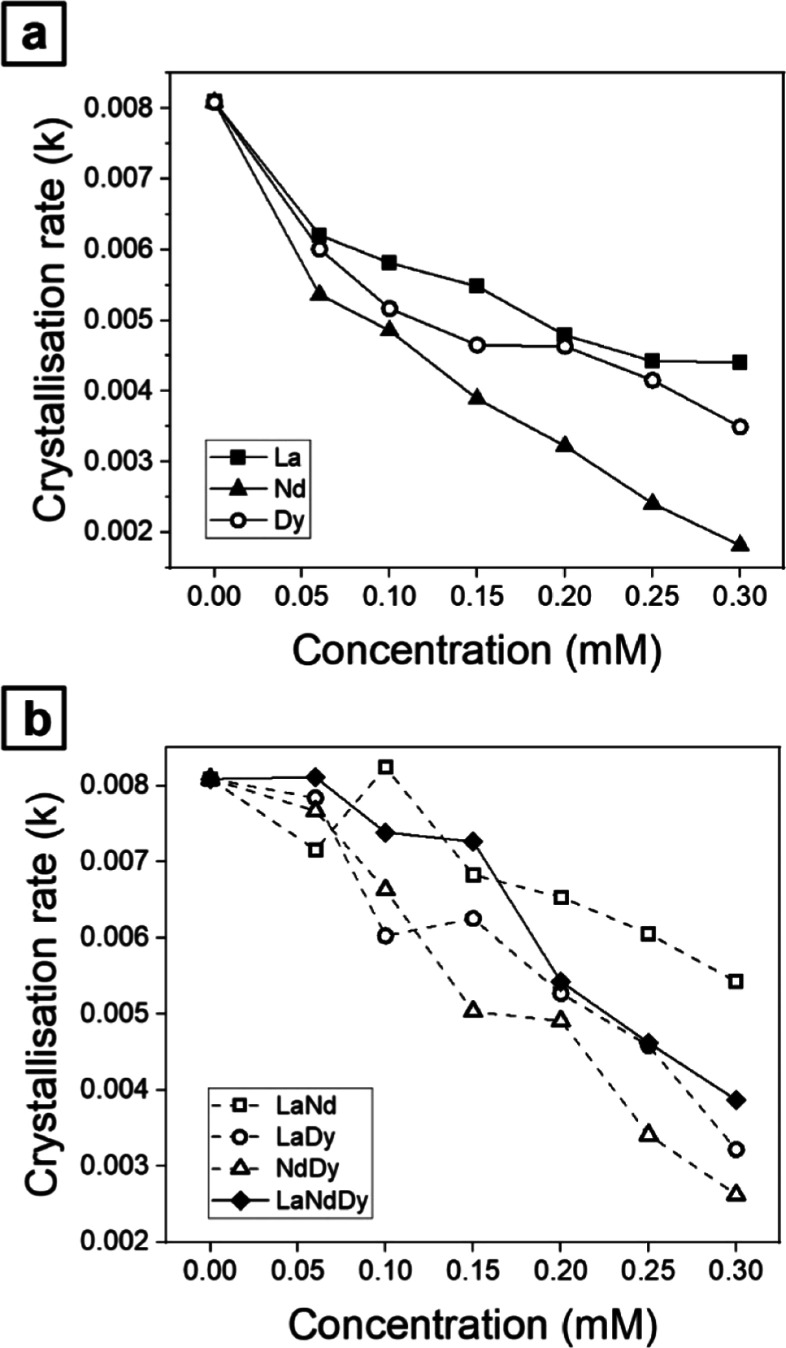
Comparison of the crystallization rates (*k*) for (a) single-REEs experiments and (b) multi-REEs experiments. In both cases *k* decreases proportionally with the increase of the concentration and the atomic mass of REEs in the system.

The initiation of CaCO_3_ crystallization experienced a delay at various concentrations of REEs (up to 0.3 mM, either alone or in combination; Figures 2 and 3). The delay in the induction time was found to be proportional to the REEs/Ca ratio in the initial solution and the atomic mass of the REEs. For the pure (0 mM) experiment, the induction time was approximately 20 s, which extended nearly 1 order of magnitude longer for the 0.3 mM Nd system (∼150 s), exhibiting the longest induction period among all experiments. The completion of the primary crystallization was achieved within a time range spanning from 340 s in the pure system to 930 s for the 0.3 mM Nd system. Overall, single-REEs experiments showed that the highest crystallization rate happened in the La system, followed by Dy and Nd (Figure 11a). However, the experiments with different combination of REEs revealed that calcite crystallization rates were the fastest in the La + Nd experiments, followed by La + Dy and then Nd + Dy. Similarly, the experiment with the three REEs combined together show a trend that was approximate the average of the experiments with two REEs (Figure 11b). In all these experiments, the crystallization rate decreased proportionally to the concentration of each specific element(s) in the starting solution, and generally, this is quantified in around half an order of magnitude.

In general, our findings align with previous studies that have documented the inhibitory effect of foreign ions and organic compounds on the crystallization of calcite, with the degree of inhibition being directly proportional to their concentration, like Mg^2+^,^44^ citrate,^67^ fluoride,^71^ ethanol^72^ and La^3+^.^47,48^ These two last studies, in particular, along with Akagi and Kono (1995)^46^ are the only ones that, at the best of our knowledge, have investigated the kinetics and mechanisms of calcite growth under the influence of specific REEs (La). Our experimental findings revealed that among the single-REEs experiments in this study, Nd produced the most substantial delay in the onset of the reaction and resulted in the slowest rate of crystallization of CaCO_3_. It was originally anticipated that La would have the least impact of calcite growth rate, followed by Nd, and then Dy. This was based on the ionic potentials of the REE^3+^ ions. The REEs display a phenomenon known as the lanthanide contraction, with their ionic radii decreasing with increasing atomic number.^20^ The reduction in ionic radius coincides with an increase in their ionic potentials, calculated by dividing the ion’s valence by its ionic radius. This way, the ionic potentials for La^3+^, Nd^3+^, and Dy^3+^ are 2.60, 2.77, and 3.03 Å^–1^, respectively.^49^ Compared to the ionic potentials of the REEs considered in this work, the ionic potential of Ca^2+^ is 2.02 Å^–1^.^49^ This means that it is slightly larger than that of La^3+^ (1.60 Å^–1^) and smaller than those of Nd^3+^ (2.77 Å^–1^) and Dy^3+^ (3.03 Å^–1^). For comparison, the ionic potential of Mg^2+^ is 3.07 Å^–1^,^49^ larger than Ca^2+^, La^3+^, and Nd^3+^ and close to Dy^3+^.

A higher ionic potential is translated into a greater energy requirement for the dehydration of REE^3+^ ions in solution before they are sorbed to mineral structures. This is an effect observed in the crystallization of REEs carbonates from solution and in replacement experiments (e.g., refs (49), (55), (62), and (65)) as well as in the sorption of divalent ions on calcite (e.g., refs (73) and (74)). Interestingly, when the crystallization rates of CaCO_3_ under the influence of two REEs are compared, their behavior is consistent with the average of their ionic potentials combined. This is La + Nd (2.69 Å^–1^), La + Dy (2.82 Å^–1^), and Nd + Dy (2.90 Å^–1^). Similarly, the experiments with the three REEs combined together show a trend comparable with the experiments with two REEs, consistent with the average value of the ionic potentials (2.81 Å^–1^) of these three ions.

However, our data revealed that Nd, when used alone, exerted the greatest influence on CaCO_3_ crystallization, despite having a lower ionic potential than Dy. It is known that REEs can adsorb and incorporate in the structure of calcite, even after short reaction times.^37,39^ One plausible explanation for the observed behavior of Nd (compared to Dy) could be attributed to the distinct coordination of different REEs within the octahedral Ca site during coprecipitation with calcite, as reported by Elzinga et al. (2002).^75^ In their work, extended X-ray absorption fine structure spectroscopy (EXAFS) was applied to determine the local coordination of representative LREEs such as Nd and Sm and HREEs including Dy and Yb, during coprecipitation with calcite from aqueous solutions at ambient temperature. The results confirmed the substitution of REEs in the Ca sites but also highlighted a discrepancy in the first shell REE-O distances in calcite compared to the Ca–O distances, with LREEs exhibiting longer distances and HREEs showing shorter distances. Specifically, the reported distance was consistent with a 7-fold coordination for LREEs and a 6-fold coordination for HREEs. The observed difference in coordination may provide an explanation for the moderate effects exhibited by Dy compared to the more pronounced impact of neodymium Nd on CaCO_3_ crystallization rates in our study.

While there is clear spectroscopic evidence of the incorporation of REEs into the Ca^2+^ site in calcite,^75,76^ the charge compensation mechanism is still unknown. The incorporation of trivalent REEs in place of divalent Ca is challenging, as it results in an excess charge. Early studies inferred that the excess charge is compensated by monovalent ions or vacancies.^34^ However, recent studies have shown that heterovalent substitution is more complex. EXAFS spectroscopy studies demonstrated that Nd and Sm in calcite adopt 7-fold coordination, while heavier REEs such as Dy and Yb exhibit the same 6-fold coordination as Ca.^75,76^ The increase in the coordination number was attributed to the formation of a bidentate bond between the REE^3+^ and a CO_3_^2–^ group, which alone cannot balance the charge. The EXAFS data could be explained by the presence of partially hydrated REEs species, allowing the formation of a bidentate bond between REE^3+–^ OH^–^ and CO_3_^2–^ groups, which would balance the charge and explain both the bonding mechanism and the charge balance.

Also, it is worth mentioning another possible explanation that would involve the formation of an REEs-rich amorphous precursor phase. A tiny increase in the turbidity (corresponding to α ≃ 0–0.015) was observed a few seconds after the mixing of the solutions and before starting to follow the classic sigmoidal pattern in the highest concentration experiments (e.g., 0.25, 0.3 mM Nd, Dy or Nd + Dy; Figures 2 and 3) at the very early stages of the reaction for a few (<100) seconds. We interpret them as an indication of the possible formation of an amorphous transient precursor phase prior to the crystallization of the CaCO_3_. The formation of this transient and poorly ordered phase would be consistent with our SEM observations (Figure 9) and could have lowered the REE^3+^, CO_3_^2–^, and Ca^2+^ concentrations in the aqueous solution, contributing to an increase in the crystallization rate of CaCO_3_ in the experiments using the heaviest REEs (Dy) of the group.

In the pure system, CaCO_3_ crystallized as 95 wt % of calcite and only 5 wt % of vaterite, while in the presence of REEs, the vaterite seemed to be favored over calcite. It is not possible to clearly infer a general trend in the results, but it is evident that vaterite wt % is generally lower at lower concentrations (0.06 mM) than at higher concentrations (0.3 mM) in the single-REEs experiments. On the contrary, the behavior in the multi-REEs experiments is diametrically opposite. Overall vaterite wt % is greater in the multi-REEs experiments compared to the single REEs. We suggest that the lowering of the REE^3+^/Ca^2+^ ratio due to the formation of a poorly ordered phase could also have affected the formation of vaterite, potentially accounting for the observed variations in the weight percentage of this polymorph. The concentration of carbonate ions in the system plays a crucial role in the kinetics of CaCO_3_ crystallization as well as the formation of the amorphous phase. In the scenario where CaCO_3_ crystallization occurs prior to the development of the amorphous REEs carbonate phase, the concentration of carbonate ions in the system will undergo a rapid decline, subsequently reducing the supersaturation levels for alternative REEs carbonate phases, delaying or impeding its formation. However, if an REEs carbonate amorphous phase forms prior to the nucleation of any CaCO_3_ polymorphs, the concentration of REE^3+^, Ca^2+^, and CO_3_^2–^ ions in solution will decrease, also decreasing the supersaturation levels for calcite and vaterite and consequently affecting their crystallization kinetics.

In our experiments in the pure system, both vaterite and calcite showed their usual spherulitic and rhombohedral morphologies. When crystallized in the presence of REEs the crystals developed different degrees of imperfections and defects, varying as a function of differences in the concentration or combinations of the REEs ions. The defects observed in our experiments, consisting of holes with variable diameters (0.5 to 1 μm) and variations of the surface roughness, became more abundant proportionally with the concentration and atomic mass of the REEs, indicating a potential growth inhibition during the latest stages of spherulitic growth without significantly impacting the underlying mechanism of crystallization. The growth morphology of vaterite (polycrystalline spheres consisting of tiny nanoparticles) is consistent with spherulitic growth, an extremely fast crystallization mechanism that requires high supersaturation levels (SI = 2–3).^30,77−79^ Also, the wider fwhm observed in the Bragg peaks of vaterite compared to calcite in all experiments is indicative of a smaller crystallite size in vaterite. This characteristic is typical of crystals grown by spherulitic growth and is consistent with the formation of nanocrystalline aggregates in vaterite. In all of the experiments, the diameter and shapes of the spheres (2–3 μm) remained constant. However, surface defects and imperfections observed on the spheres in the experiments involving higher concentrations of heavier REEs (Figure 6) seem to have occurred during the later stages of spherulitic growth crystallization, when the supersaturation levels were comparatively lower.

These morphological changes are relatively minor compared to those reported in other studies involving vaterite crystallization under various concentrations of organics, inorganics, and different physicochemical conditions, which were translated into other morphological forms like hexagonal crystals,^80,81^ spindles,^82^ lamellar,^83^ flower-like,^84^ rosettes^80^, and eventually microtablets.^85,86^

The influence of REEs on the morphology of calcite was considerably more pronounced. Similarly to vaterite, the size of calcite crystals (3–4 μm) did not change significantly in the presence of REE ions. However, at higher concentrations of REEs, the substantial variation in crystal morphology, transitioning from the characteristic rhombohedral shape in the pure system to crystals displaying extended defects, was comparable to the morphologies reported by Paquette et al. (1996),^87^ Zhang and Dawe (2000)^88^ and Kim et al. (2017).^89^ These studies report irregular surfaces observed along specific edges and corners of calcite crystals when exposed to ions, such as Mg^2+^, SO_4_^2–^, and PO_4_^3–^ combined with Mg^2+^. In our experiments, lower concentrations and lower atomic masses of REEs resulted in minor defects observed at the edges and corners of the crystals. However, these conditions promoted the growth of nearly perfect rhombohedral crystals, indicating that the solids can be considered as single crystals. However, at higher concentrations and atomic masses of REEs, the surface of calcite underwent a significant transformation, transitioning from a smooth texture to a highly irregular and rough surface, with multiple defects at the edges and corners of the crystals. The morphology of these crystals, sometimes consisting of a single crystal core encased in a thin polycrystalline shell, indicates that calcite does not seem to fully grow via a classical route (from a single nucleus). This is also evidenced by its surface architecture (including edges and corners), consisting of many nanodomains (∼100 nm) stacked on top of each other, which often tend to be growth-oriented following the [010] direction on the (104) face (Figures 7 and 8).

The model developed by Zhang and Dawe (2000)^88^ provides valuable insights into understanding the crystallization morphology of calcite in the presence of REEs observed in our experiments. Their work on the effect of Mg^2+^ on calcite crystallization kinetics and morphology suggests that this ion inhibits calcite growth by nonuniformly adsorping into different surface sites of the calcite crystals,^88,90^ resulting in the development of new surfaces with higher Mg^2+^ density and slower growth rates compared to the original surfaces. The model classifies crystal surfaces into three groups: F (flat), S (stepped), and K (kinked).^88,91,92^ As Mg^2+^ has a higher affinity for some of these sites, its adsorption and dehydration prior to structural incorporation preferentially delay growth in specific directions like edges and corners, leading to the development of different crystal morphologies. The K and S surfaces are characterized by an abundance of kinks and steps, respectively, and exhibit lower growth rates compared to the F surface. These K and S surfaces are typically absent in equilibrium crystal morphologies.^88,91^

As REE^3+^ has a very strong affinity for calcite,^34,35^ we propose a crystallization model where these ions adsorb onto different calcite surfaces with varying affinities and densities prior to their incorporation into the crystal structure. This preferential adsorption would inhibit crystal growth on specific surfaces, such as edges and corners, resulting in complex morphologies beyond the classical rhombohedral shape. The similar behavior of Mg^2+^ and REE^3+^ during calcite crystallization may be attributed to their strong hydration shells, especially the REE^3+^ ions, which are considerably larger and stronger compared to Ca^2+^. Additionally, our findings demonstrate that a lighter REEs like La^3+^ has a narrower impact on the crystallization of calcite, whereas heavier REEs like Nd^3+^ and Dy^3+^ exert a stronger influence. This correlation aligns with the fact that ions with smaller radii possess a higher ionic potential and, therefore, stronger hydration shells, leading to a higher proportion of defects in calcite crystals, as observed in our SEM images.

## Conclusions

Our findings provide novel insights into the influence of REEs on the crystallization of CaCO_3_ from solution. The presence of REEs ions in the aqueous solution exerts notable effects on the kinetics, mechanisms, and morphologies of the CaCO_3_ polymorphs. These effects are primarily governed by the number, concentration, and atomic mass of REEs present in the aqueous solution.

REEs significantly reduce the crystallization rate of CaCO_3_. In single-REEs experiments, Nd exhibits the strongest effect, followed by Dy and eventually La. In multi-REEs experiments, the effects correlate with the average ionic potential of the ions used. In all cases, vaterite and calcite are the main polymorphs formed in the presence of REEs. Relative to the pure system the two polymorphs show significant variations in their wt % and vaterite formation is favored over calcite. Both CaCO_3_ polymorphs exhibit growth defects, and calcite displays very pronounced irregularities, such as rough surfaces, edge and corner distortions, and the occurrence of nanodomains on the (104) face, very likely as a result of the adsorption of REEs onto different calcite surfaces with varying affinities and densities before incorporating into the mineral structure. This preferential adsorption inhibits crystal growth on specific surfaces, leading to complex morphologies. This phenomenon is likely a consequence of the stronger hydration shells of REE^3+^ in solution due to the higher ionic potential of these ions compared to Ca^2+^. The potential formation of a poorly ordered REEs carbonate precursor phase at the early stages of the experiment prior to the crystallization of CaCO_3_ can also decrease the effective concentration of REE^3+^ in solution, affecting the kinetics of crystallization and crystal morphology.

Our results provide valuable information about the behavior of these elements in natural systems containing CaCO_3_ minerals and REEs-bearing fluids and can contribute to the advancement of REEs extraction and recovery processes, addressing the increasing demand for these valuable metals.

## Supporting Information Available

(1) Quantification values of the wt % of crystalline phases carried out with the Rietveld refinement software TOPAS. (2) Points of analysis obtained both on CaCO_3_ polymorphs (calcite and vaterite) and on a homogeneous amorphous Nd- and Ca-bearing phase in an experiment with 0.3 mM of Nd. (3) SEM picture showing the location of points of analysis. (4) Graph showing the *k*_1_ of the single REE experiments. (5) Graph showing the *k*_2_ of the single REE experiments. (6) Graph showing the *k*_1_ of the multi-REE experiments. (7) Graph showing the *k*_2_ of the multi REE experiments. (8) Graph showing the wt % of calcite and vaterite obtained in all the single and multi-REEs experiments. (9) XRD patterns showing two samples in which it is possible to identify the absence (La 0.06 mM, above) and the presence (La + Nd + Dy, below) of humps that correspond consistently to poorly ordered precursor phases. (10) Powder XRD patterns of the pure system (0 mM REEs) and of the experiments in the presence of La + Nd + Dy both at low (0.06 mM) and high (0.3 mM) concentrations (PDF)
