# Supplementary material for: Impact of Rare Earth Elements on CaCO3 Crystallization: Insights into Kinetics, Mechanisms, and Crystal Morphology
Source: Cryst Growth Des. 2023 Dec 30;24(2):632–45. doi: 10.1021/acs.cgd.3c00858 (PMC10797595; doi:10.1021/acs.cgd.3c00858)

Impact of rare earth elements on $CaCO_3$ crystallisation: Insights into kinetics, mechanisms, and crystal morphology

Luca Terribili[1]*, Remi Rateau[1], Adrienn M. Szucs [1], Melanie Maddin[1], Juan Diego Rodriguez-Blanco[1]*.
* terribil@tcd.ie, J.D.Rodriguez-Blanco@tcd.ie
1 Department of Geology. School of Natural Sciences. Trinity College Dublin, D02 PN40, Ireland

SUPPORTING INFORMATION FOR THE PAPER

Table SI-1. Quantification values of the wt% of crystalline phases carried out with the Rietveld refinement software TOPAS. The values displayed refer to the Pure system (0 mM of REEs) as well to the lower and higher concentration experiments for every REE and combination of REEs used.

REE	Concentration	Calcite	Vaterite
**Pure System**	0 mM	95%	5%
**La**	0.06 mM	60%	40%
**La**	0.3 mM	31%	69%
**Nd**	0.06 mM	68%	32%
**Nd**	0.3 mM	64%	36%
**Dy**	0.06 mM	64%	36%
**Dy**	0.3 mM	39%	61%
**LaNd**	0.06 mM	46%	54%
**LaNd**	0.3 mM	62%	38%
**LaDy**	0.06 mM	40%	60%
**LaDy**	0.3 mM	42%	58%
**NdDy**	0.06 mM	52%	48%
**NdDy**	0.3 mM	72%	28%
**LaNdDy**	0.06 mM	31%	69%
**LaNdDy**	0.3 mM	32%	68%

Table SI-2. Points of analysis obtained both on $CaCO_3$ polymorphs (calcite and vaterite) and on a homogeneous amorphous Nd- and Ca-bearing phase in an experiment with 0.3 mM of Nd. In Fig SI-1 is showed the exact location where the points of analysis were carried out.

**Point of analysis**	**Atomic %**		
	**Oxygen**	**Calcium**	**Neodymium**
**Spectrum 16**	79.22	19.57	1.21
**Spectrum 17**	81.15	18.50	0.35
**Spectrum 18**	78.24	20.94	0.81
**Spectrum 19**	68.39	21.85	9.76
**Spectrum 20**	68.64	17.90	13.45
**Spectrum 21**	77.19	15.55	7.26
**Spectrum 22**	66.75	24.90	8.35
**Spectrum 23**	83.53	14.31	2.18
**Spectrum 24**	71.14	21.91	6.95
**Spectrum 25**	72.86	17.54	9.60
**Spectrum 26**	60.08	22.19	17.73
**Spectrum 27**	58.34	24.54	17.12
**Spectrum 29**	84.40	14.78	0.82
**Spectrum 30**	81.00	17.67	1.33
**Spectrum 31**	84.83	14.46	0.71
**Spectrum 32**	62.13	23.71	14.16
**Spectrum 33**	71.79	17.20	10.99
**Spectrum 34**	67.16	17.47	15.39
**Spectrum 35**	62.34	22.71	14.95
**Spectrum 36**	79.86	19.01	1.13
**Spectrum 37**	80.29	18.82	0.87
**Spectrum 38**	84.91	13.73	1.36

Fig SI-1. SEM picture showing the location of points of analysis displayed in Table SI-2.

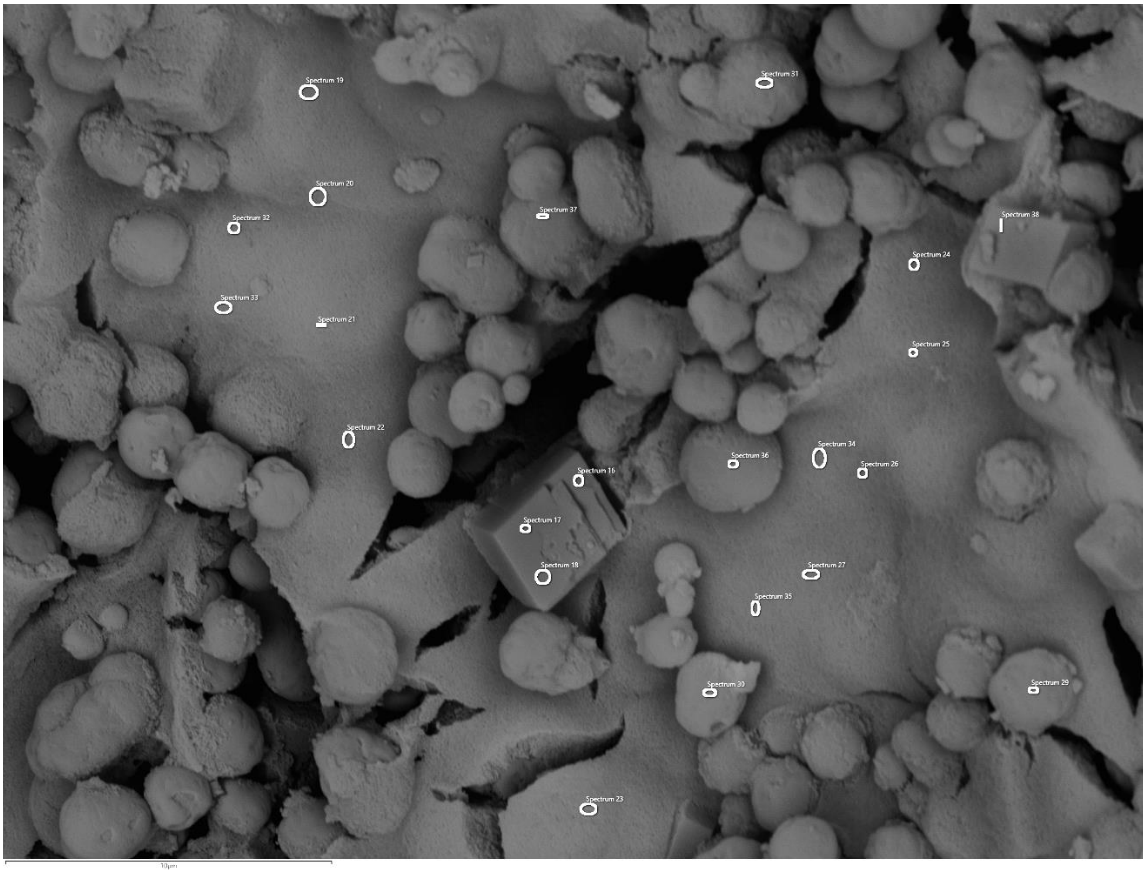

Fig SI-2. Graph showing the k1 of the single REE experiments.

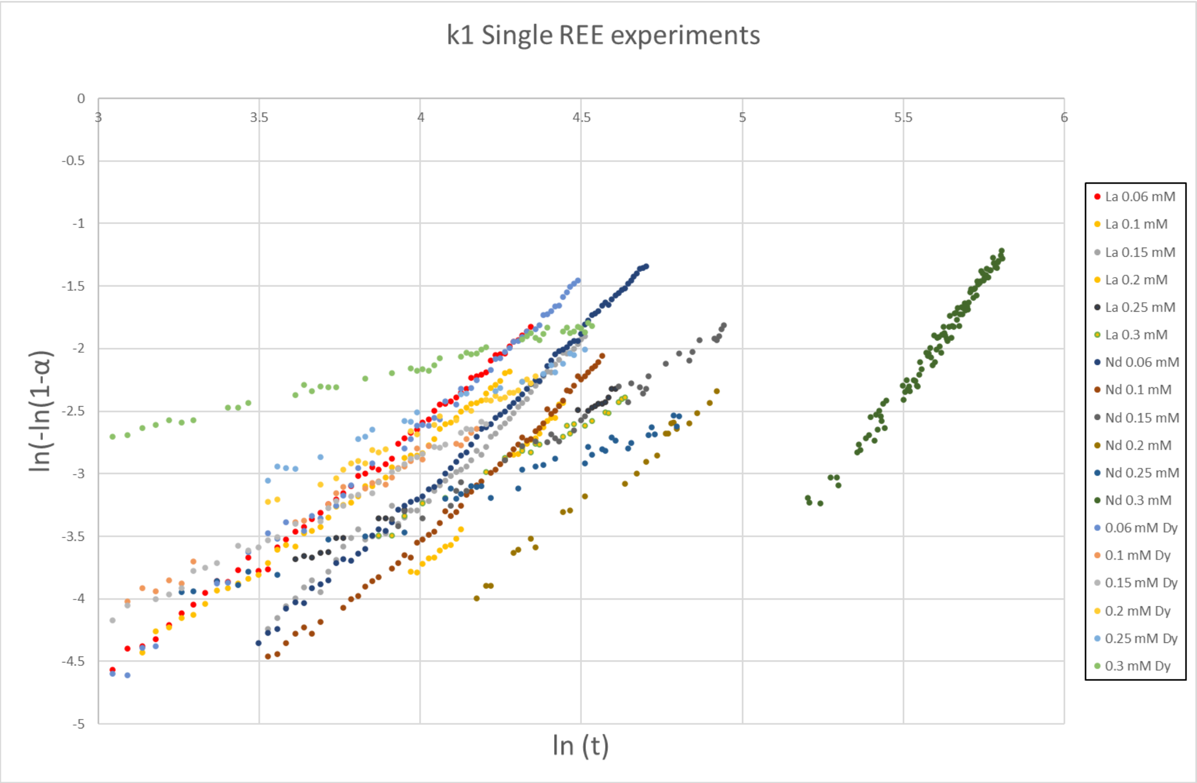

Fig SI-3. Graph showing the k2 of the single REE experiments.

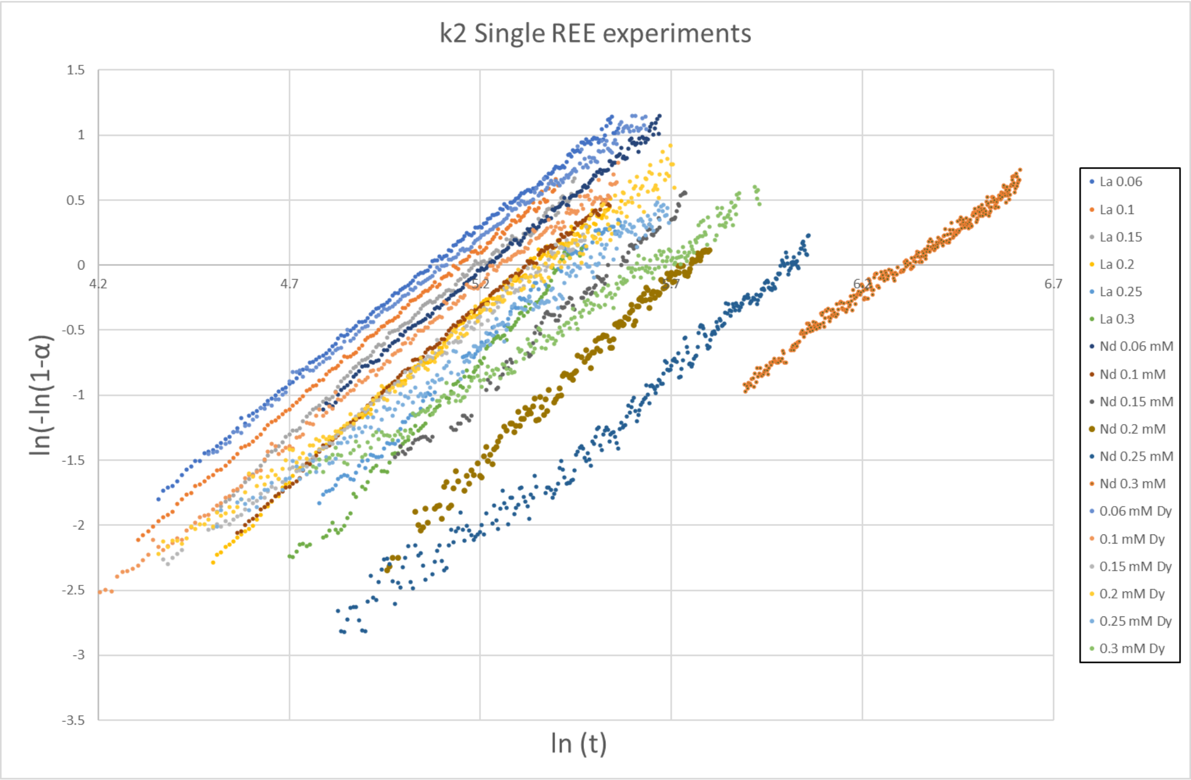

Fig SI-4. Graph showing the k1 of the multi REE experiments.

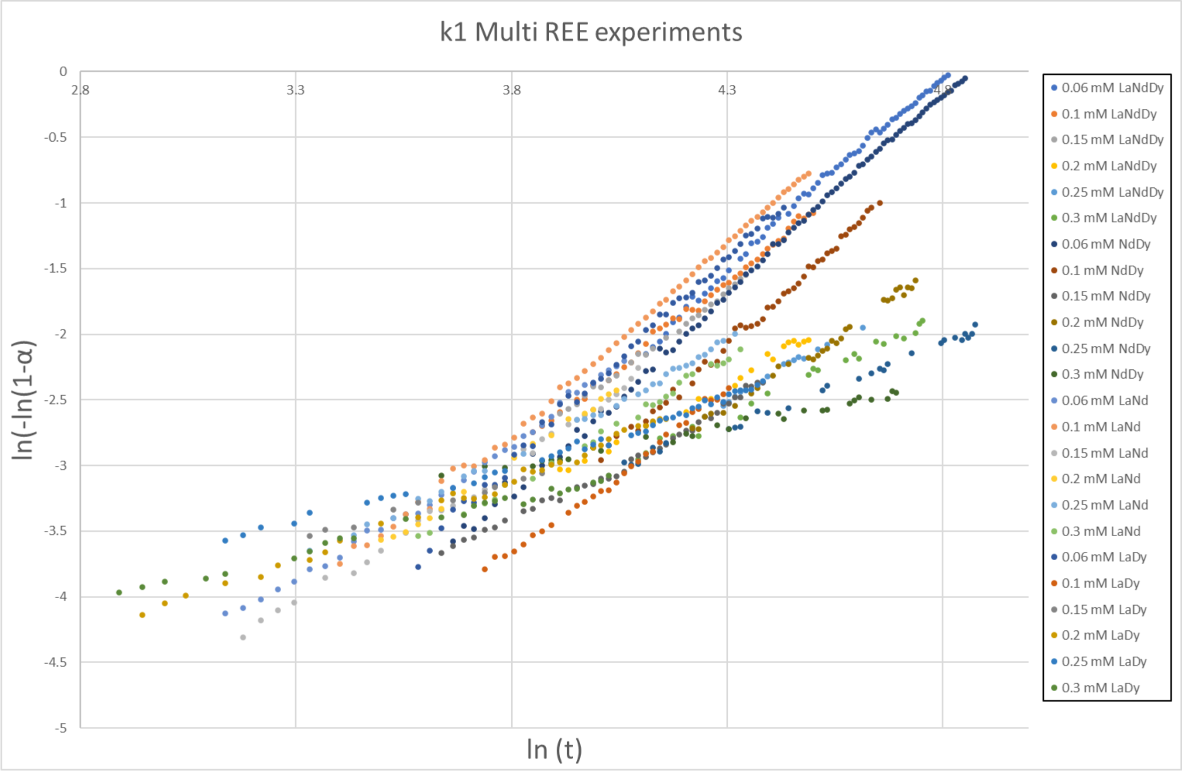

Fig SI-5. Graph showing the k2 of the multi REE experiments.

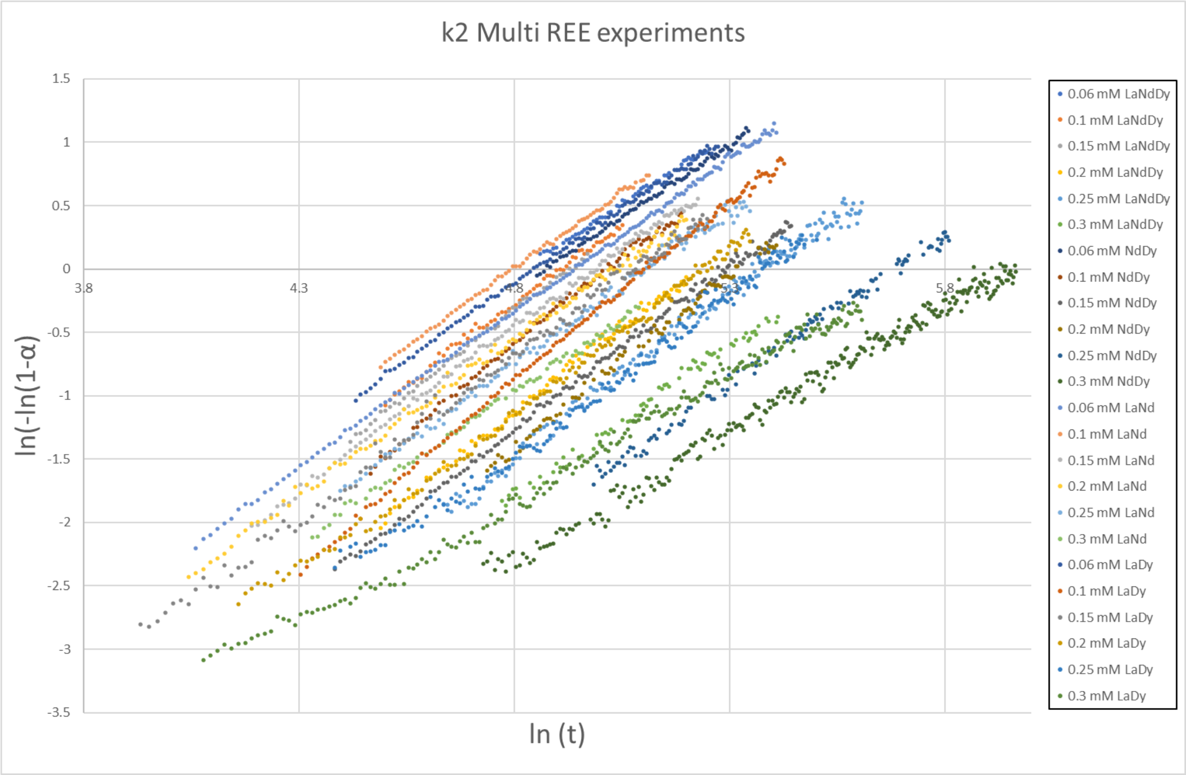

Fig SI-6. Graph showing the wt% of calcite and vaterite obtained in all the single and multi-REEs experiments.

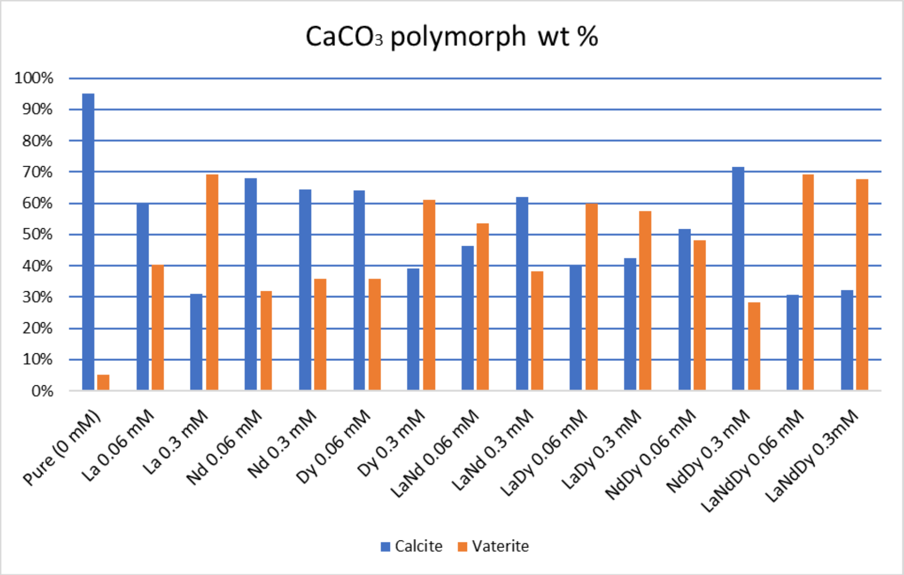

Fig SI-7. XRD patterns showing two samples in which it is possible to identify the absence (La 0.06 mM, above) and the presence (La+Nd+Dy, below) of humps that correspond consistently to poorly-ordered precursor phases.

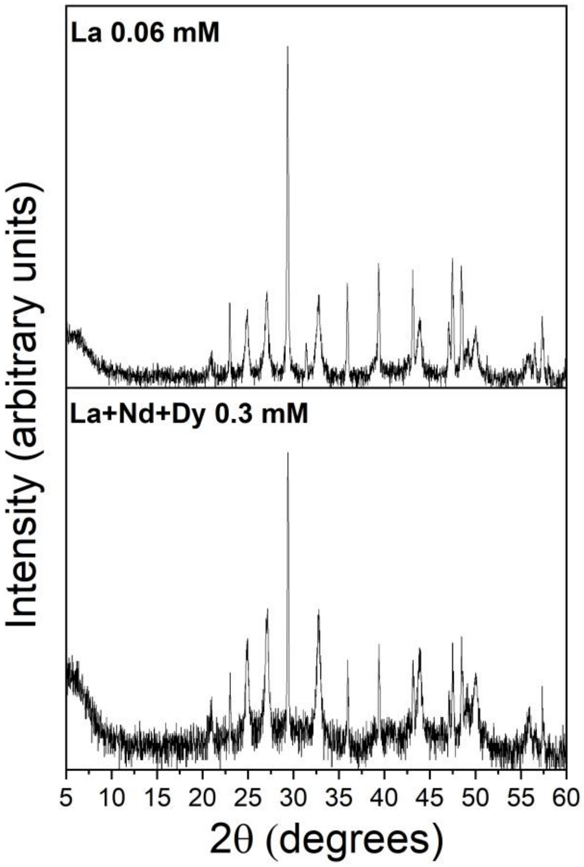

Fig SI-8. Powder XRD patterns of the pure system (0 mM REEs) and of the experiments in the presence of La+Nd+Dy both at low (0.06 mM) and high (0.3 mM) concentration. The small square next to the pure system pattern is a zoom between 20 and 35 2θ degrees in order to show better the presence of the vaterite peaks.

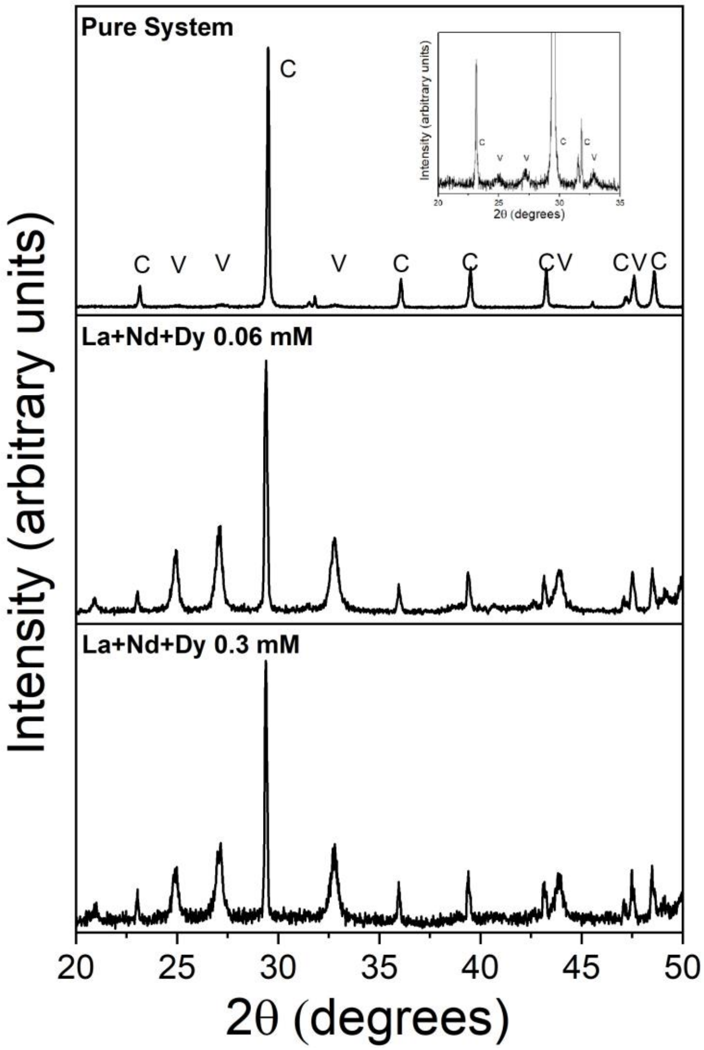

Supplement: Supplementary file 1 — cg3c00858_si_001.pdf [file cg3c00858_si_001.pdf]
